# Synthesis of Monolithic Potassium Geopolymer Ceramics Assisted by Molten Salt

**DOI:** 10.3390/ma12030461

**Published:** 2019-02-02

**Authors:** Tao Ai, Feng-hua Hong, Yi-na Kang, Hao-ran Zhang, Xin Yan

**Affiliations:** 1 School of Materials Science & Engineering, Chang’an University, Xi’an 710061, China; solarannn@163.com (H.Z.); xinyan@chd.edu.cn (X.Y.); 2 Sinosteel Luoyang Institute of Refractories Research Co., Ltd., Luoyang 471039, China; kangyn@163.com

**Keywords:** potassium geopolymer, leucite, molten salt, ceramics, sintering

## Abstract

Potassium geopolymer (KGP) ceramics are synthesized by the molten salt method. Metakaolin changes to a potassium geopolymer through a reaction with potassium silicate at 80 °C/24 h. Potassium geopolymer, with a silicon to aluminum (Si/Al) molar ratio of 2, turns into a leucite ceramic in molten potassium salt (KCl) at 900 °C/6 h. X-Ray Diffraction analysis of the KGP treated by molten KCl salt shows the amorphous phase change to leucite crystal phase. A scanning electron microscope image of sintered KGP proves that the grain size of the leucite crystal decreases with soaking time. Compared with solid state sintering, liquid molten salt sintering KGP can be more easily formed into dense ceramics at lower temperatures.

## 1. Introduction

A geopolymer is a special, high performance inorganic polymer material with an amorphous structure [1,2]. Because of the special inorganic poly-condensation of three-dimensional oxide network structure, geopolymers have an excellent fire resistance, low cost, are environmentally friendly and have great thermal properties [3].

Because of these properties KGP is an important geopolymer. It can be converted to tetragonal or cubic leucite (K_2_O·Al_2_O_3_·4SiO_2_) ceramics after being treated at high temperatures [4]. Leucite ceramics are useful for materials with high accessional values, such as dental porcelains, refractories, and structural ceramic materials [5,6,7,8,9]. However, due to large capillary forces acting on capillary channels during the high temperature treatment, surface cracking of the monolithic geopolymer ceramics results [10].

Molten salt is often used as a reaction media to increase the crystallinity of materials and effectively control the structure of crystals at relatively low temperatures [11,12,13]. The production of geopolymer ceramics with the help of molten salt has not previously been reported.

In this paper, Potassium geopolymer ceramics were prepared by the molten salt method at 900 °C. The effects of the molten KCl salt and the calcination time on the microstructure and the physical properties of the resulted products were systematically studied.

## 2. Materials and Methods

### 2.1. Synthesis of Potassium Geopolymer

First a potassium silicate solution (K_2_O·2SiO_2_·15H_2_O) was prepared by dissolving amorphous silica into the KOH solution (solid/liquid = 1). The solution was stirred continuously at 40 °C. The mixed solution was allowed to mature under stirring for 12 h in order to completely dissolve the silica. Kaolin powders were then calcined at 750 °C for 2 h, in order to produce the metakaolin (Al_2_O_3_·2SiO_2_) powders. A measured quantity of the metakaolin was added to the potassium silicate solution (solid/liquid = 1) and stirred for 30 min to allow it to adequately react. The resulting mixture was cooled to room temperature and left there for 12 h. After cooling the mixture was pressed into a round sheet about 33 mm in diameter and about 3 mm thick. This was then cured at 70 °C for 48 h. After demolding, it was dried at 70 °C for 2 h.

### 2.2. Synthesis of Leucite Ceramics

The monolithic KGP was ground into a powder. The powder was placed into an alumina crucible and mixed with KCl salt. The composition ratio of the KGP/KCl mixture is 1/10. Three samples were produced and each was heated in a stove at 900 °C for periods of 4 h, 6 h, and 8 h. After cooling to room temperature each ceramic powder sample was washed with hot distilled water to remove the residual salt. The resulting leucite ceramic powder was then dried at 80 °C for 24 h. The resulting leucites produced at 900 °C for 4 h were labled as LC4, and the resulting leucites produced at 900 °C for 8 h were labeled LC8 and LC8, and 8 h 900 °C heating times.

In order to highlight the effect of molten salt on ceramization of geopolymers another set of samples of KGP powder was sintered at 900 °C and 1200 °C without KCl salt as a control.

### 2.3. Preparation of Monolithic Geopolymer Ceramics

The monolithic potassium geopolymer samples were sintered in air flow with KCl molten salt at 900 °C for 4 h, 6 h, and 8 h. Another set of samples were sintered without KCl salt at 1200 °C.

The resulting samples were washed repeatedly with hot distilled water after cooling, in order to remove the residual KCl salt. All of the resulting monolithic geopolymer ceramics were dried at 80 °C for 24 h.

### 2.4. Characterization

The crystalline structure of the ceramic samples was examined by powder X-ray diffraction (XRD) Bruker D8 Advance, Cu-Kα radiation, Bremen, Germany. The surface morphology and the microstructure of the powders or fracture block of leucite ceramic specimens were investigated by using a scanning electron microscope (SEM, JSM-6460LV, JEOL, Tokyo, Japan). Gold coating was applied to these samples so that the crystalline structure could be examined by a 5.0 kV SEM. Six monolithic KGP ceramic samples were prepared. The samples were 20 mm in diameter and 20 mm in height. Their densities were determined by the Archimedes method of using density balance. Their compressive strengths were determined by a 100-ton press.

## 3. Results and Discussions

**Figure 1:**
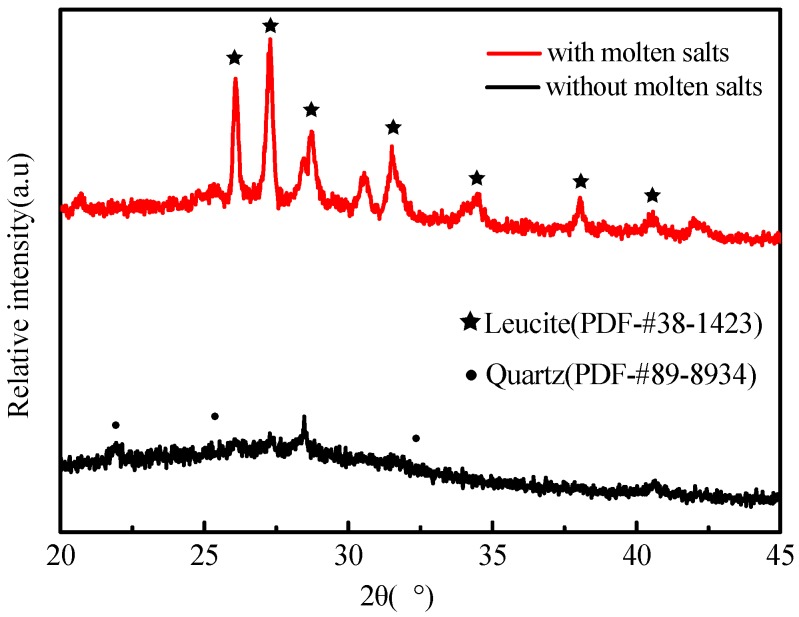
The XRD pattern of KGPs with and without molten salt.

Figure 1 shows the XRD patterns of the KGP samples after being treated at 900 °C for 4 h. A small amount of quartz appears when the KGPs are sintered without the assistance of KCl salt.

This analyses shows that the KCl also contributes to the generation of leucite. By using molten KCl salts and geopolymer ceramiztion the leucite was fully stabilized.

**Figure 2:**
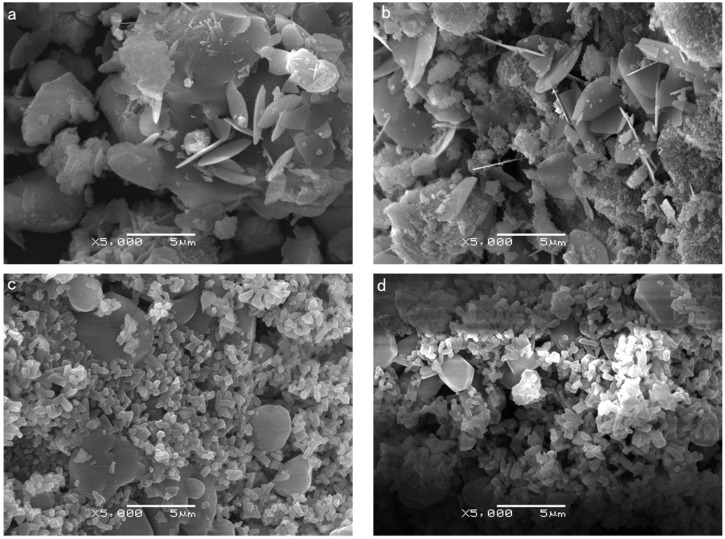
SEM images of KGP ceramics. (**a**) 1200 °C, 4 h, without KCl salt; (**b**) 1200 °C, 8 h, without KCl salt; (**c**) 900 °C, 4 h with salt (LC4); (**d**) 900 °C, 8 h with salt (LC8).

Figure 2a,b show the SEM micrograph of the KGP resulting from the 1200 °C treatment for the 4 h and 8 h periods respectively. It is observed that after treatment at 1200 °C all KGP samples developed a smooth and average flaky sheet, the amorphous phase was almost transformed to the crystalline phase while the grain size changed little. Figure 2c,d are the SEM micrograph of the KGP powder sintered with KCl molten salt at 900 °C for the 4 and 8 h periods. These images show short and small cylindrical microstructures. The size of the resulting samples was much smaller than the samples created without the molten salt. This shows that densely structured ceramic powders can be formed at lower temperatures if molten KCl salt is used.

The microstructure of leucite ceramics is composed of leucite crystal and amorphous inorganic polymer. Leucite crystals are the reinforced particles, and amorphous inorganic polymer is the matrix. The mechanical properties of leucite ceramics depend on the size and distribution of the reinforced particles. Compared with Figure 2a,b, it can be seen that in Figure 2c,d that the grain size obtained by molten salt is smaller and that there is a more uniform distribution of the leucite crystals. Fine grain and uniform distribution are useful mechanical properties of KGP ceramics.

**Figure 3:**
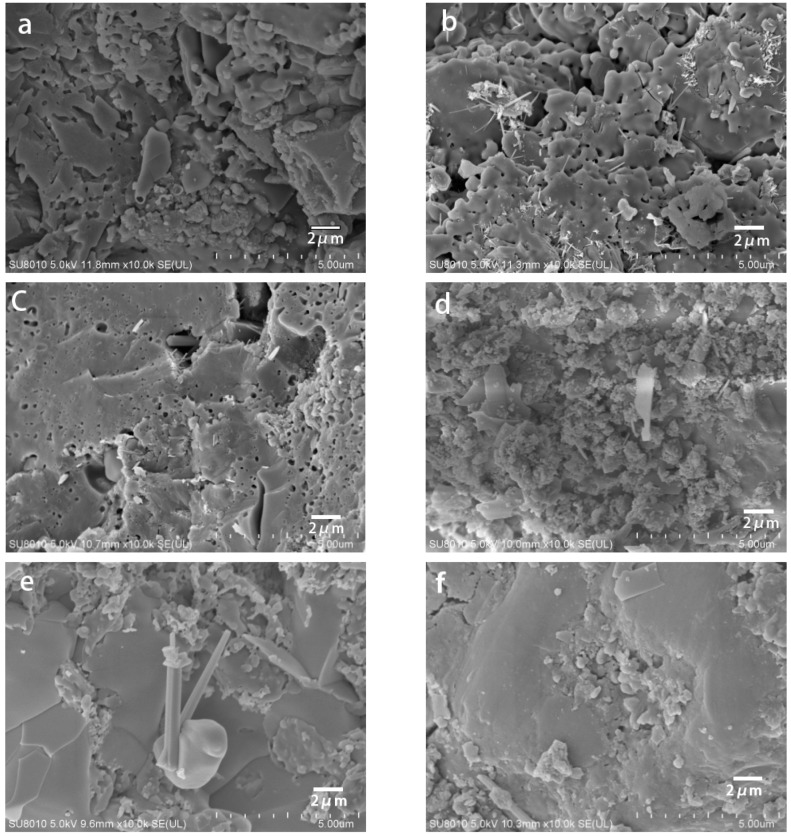
SEM images of Surface morphology of monolithic geopolymer ceramics; (**a**) KGPs 1200 °C, 4 h, without KCl salt; (**b**) KGPs 1200 °C, 6 h, without KCl salt; (**c**) KGPs 1200 °C, 8 h, without KCl salt; (**d**) KGPs 900 °C, 4 h, with KCl salt; (**e**) KGPs 900 °C, 6 h, with KCl salt; (**f**) KGPs 900 °C, 8 h, with KCl salt.

Figure 3 is SEM micrograph of the monolithic KGP under different treatment conditions.

It can clearly be seen that with the increase of holding time, pore sizes are gradually reduced and the structure of the specimen becomes smoother and denser.

Compared with the traditional sintering method, the microstructure of the samples prepared by the molten salt method at lower temperatures is smoother.

**Figure 4:**
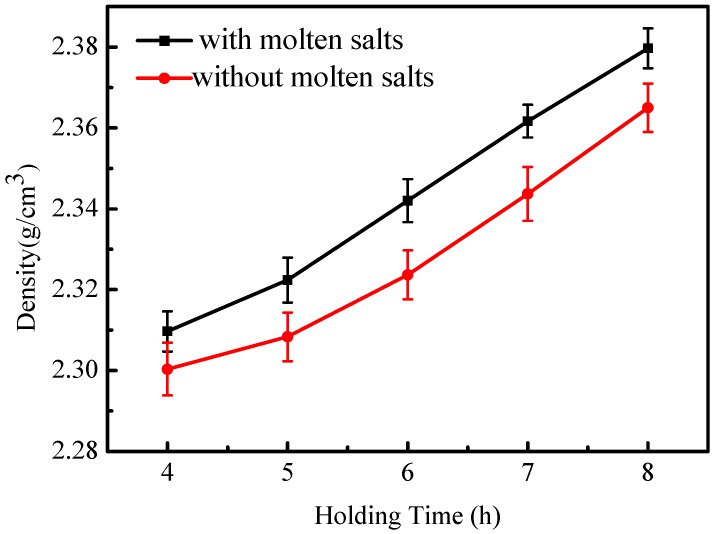
The density of monolithic geopolymer ceramics at different holding times.

As shown in Figure 4, with increased holding time, the density of the sample greatly increases. This is due mainly to the reduced porosity of the sample. Moreover, the density of the geopolymer ceramic mass which had been prepared by the molten salt method is higher than that of the conventional sample prepared at the same holding time.

**Figure 5:**
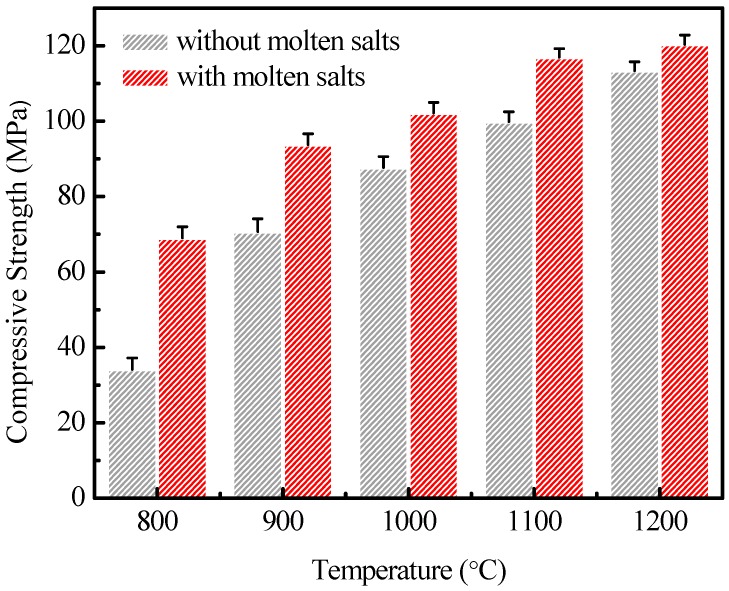
The compressive strengths of leucite ceramics produced under different sintering conditions.

Generally, the denser ceramics have higher compressive strengths. Figure 5 is the compressive strengths of leucite ceramics produced under different sintering conditions. The results show that the synthesis of leucite ceramics with the help of molten salt achieves higher compressive strengths than those samples produced without molten salt. Similarly the leucite ceramics have a higher density and finer grain size when produced with molten salt.

**Figure 6:**
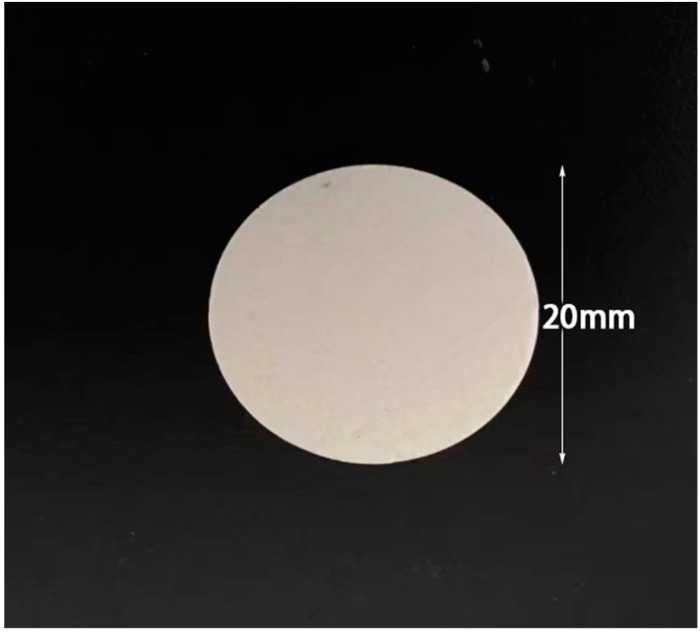
Optical photo of the monolithic leucite sample.

The shrinkage rate represents how much the size of each sample reduces after high temperature treatment. Figure 6 is the optical photo of the monolithic KGP sample. When these samples are treated by high temperatures, they will become smaller and more compact. Their sizes and shrinkage rates were measured. 

**Figure 7:**
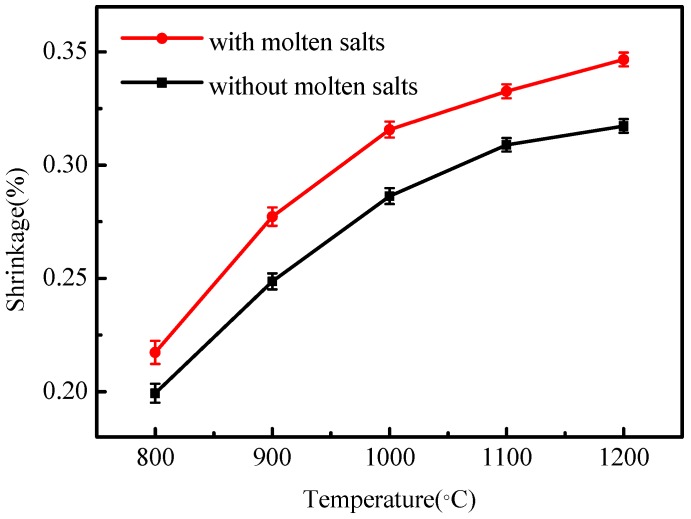
The shrinkage rate of KGP samples under different sintering conditions.

Figure 7 shows the effect of different temperatures on the shrinkage rate of KGP samples produced with and without molten salt. Because of the increased growth of leucite crystals, the shrinkage of KGP samples is higher when using the molten salt method.

The mechanism of the ceramization process of KGPcan be divided into condensation and sintering densification [1]. As the temperature increases the condensation reaction of KGP also increases. When the temperature of condensation of KGP is met, the potassium hydroxide (KOH) melts and then evaporates. When the KOH is lost the reaction proceeds toward the products. At this point the condensation get faster until the KGP changes to leucite ceramics. However, if there is not enough potassium ions provided, the de-hydroxylation will slow or even stop. This is because the potassium ions present in the molten salt promote the condensation reaction of the KGP and allow it to more easily form leucite ceramics using the molten salt method.

## 4. Conclusions

A novel process for synthesizing monolithic potassium geopolymer ceramics has been presented. Leucite ceramics synthesized using molten salt are denser and have a higher compressive strength than those produced without molten salt. The KCl molten salt also accelerates the ceramization process.
